# A Case of Unsuspected Laryngeal Atresia With Comorbid Tracheoesophageal Fistula and Cardiac Defects

**DOI:** 10.7759/cureus.56837

**Published:** 2024-03-24

**Authors:** Abigail E Reid, Swapnil Shah, Hunter Towle, Daniel Wehrmann

**Affiliations:** 1 Otolaryngology - Head and Neck Surgery, Creighton University School of Medicine, Omaha, USA; 2 Otolaryngology - Head and Neck Surgery, University of Nebraska Medical Center, Omaha, USA; 3 Otolaryngology - Head and Neck Surgery, Children's Nebraska, Omaha, USA

**Keywords:** difficult intubation, congenital anomalies, congenital high airway obstruction syndrome, tracheoesophageal fistula, laryngeal atresia

## Abstract

Laryngeal atresia is a rare congenital condition that presents with hypoxia and failed intubation attempts at birth. When diagnosed prenatally, options exist to obtain airway access during delivery. However, postnatal diagnosis requires a high degree of clinical suspicion and the prompt initiation of surgical airway management in order to avoid morbidity and mortality.

## Introduction

Congenital high airway obstruction syndrome (CHAOS) was first described by Hedrick et al. in 1994. This rare combination of features can be identified in the antenatal period by ultrasound findings of large echogenic lungs, flattened or inverted diaphragms, distal dilation of the airway, and fetal ascites/hydrops [1]. These findings are pathognomonic for complete or near-complete obstruction of the fetal upper airway and carry a poor, often fatal prognosis. Etiologies of CHAOS include laryngeal or tracheal atresia, laryngeal or tracheal webs, laryngeal or tracheal agenesis, subglottic stenosis or atresia, congenital mass, and laryngeal cyst with laryngeal atresia representing the most frequent cause [2]. Embryologically, laryngeal atresia occurs due to failed recanalization of the vestibule and subglottic regions during the development of the larynx [3]. While laryngeal atresia may present independently, it is frequently comorbid with other congenital anomalies including esophageal atresia and tracheoesophageal fistula or persistent pharyngotracheal duct which may obscure findings of CHAOS but can provide a small amount of ventilation and increase the chance of survival [4]. Regardless of comorbid findings, rapid airway establishment aided by antenatal diagnosis or a high degree of clinical suspicion postnatally is of the utmost importance in all cases of laryngeal atresia to improve outcomes.

We report the case of a premature neonate with congenital heart disease and undiagnosed laryngeal atresia with tracheoesophageal fistula. We illustrate the significant challenges associated with postnatal management without prenatal diagnosis. We also highlight the importance of having a high degree of suspicion in cases of hypoxia associated with difficulty intubating upon delivery.

## Case presentation

**Figure 1:**
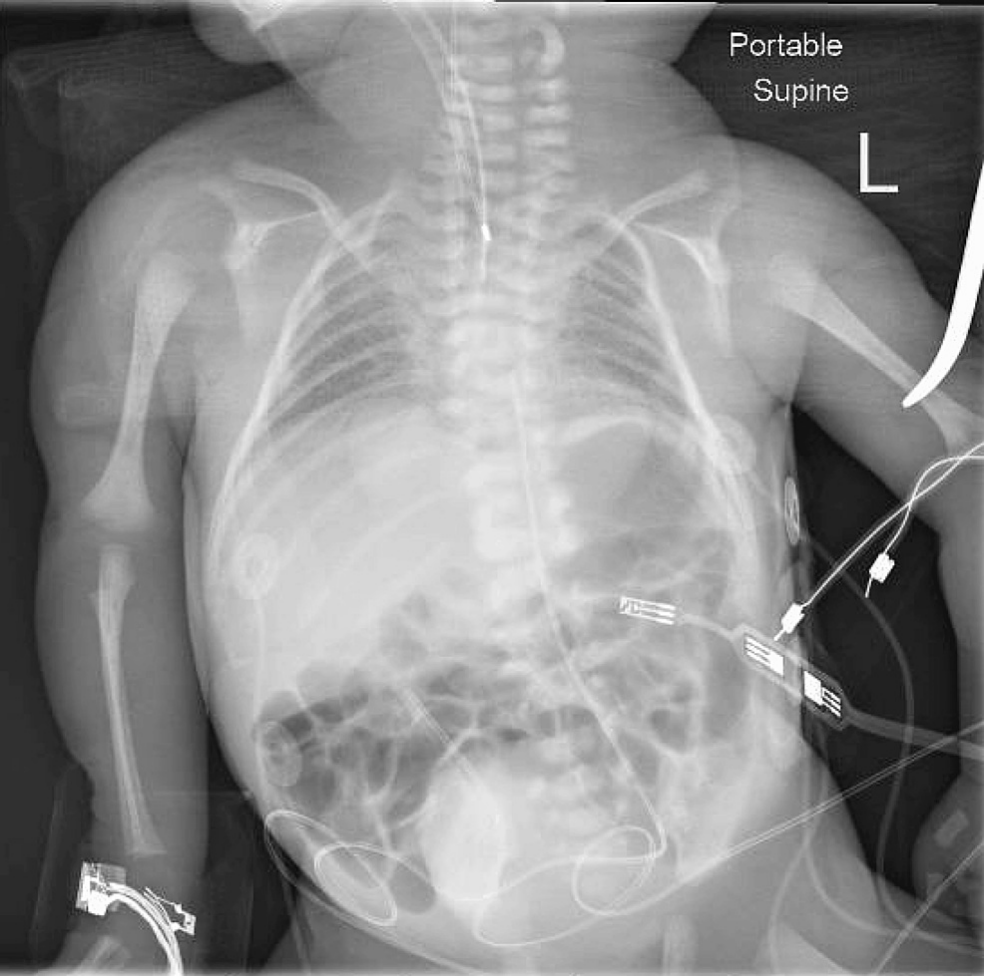
Intraoperative chest X-ray revealing air in bilateral lungs and GI tract with endotracheal tube in place

The otolaryngology service was urgently consulted to evaluate a newborn female in respiratory distress. The patient was born spontaneously at 33 weeks 0/7 days gestation via vaginal delivery to a 36-year-old mother (G2P1) with an insignificant/non-contributory past medical history who received regular prenatal care. Pregnancy was complicated by premature labor and a diagnosis of fetal congenital heart disease (CHD). Upon evaluation with a fetal echocardiogram at an outside hospital, the patient exhibited a double outlet right ventricle (DORV) with subaortic ventricular septal defect (VSD), hypoplastic and thickened pulmonary valve with small confluent branch pulmonary arteries, and a small patent ductus arteriosus (PDA). The patient’s APGAR scores at one and five minutes were two and four, respectively. The patient was successfully intubated 35 minutes postpartum on the fifth attempt. After intubation, the patient’s SpO2 remained between 50 and 60 even while on 100% FiO2 which was attributed to a large air leak. During the evaluation of the patient's airway by otolaryngology at the bedside, it was noted that the endotracheal tube was placed in the esophagus. Given this finding, the patient’s 2.5 endotracheal tube (ETT) was removed at the bedside, and re-intubation was attempted without success. This prompted an urgent assessment and re-intubation under general anesthesia to establish a safe airway. Prior to transport to the operating room, the ETT was replaced in the esophagus to maintain saturations in the 50s. In the operating room, a direct laryngoscopy and bronchoscopy demonstrated an atretic plate immediately below the vocal folds. With an established grade 1 view of the larynx, this was indicative of laryngeal atresia. Due to the presentation of bubbles at the level of the esophagus, the patient was re-intubated via the esophagus to re-establish some degree of ventilation. The parents were consulted and consented to a tracheostomy to establish an airway. During blunt dissection of the trachea, the endotracheal tube within the esophagus was visualized along the anterior wall indicating strong likelihood of a large tracheoesophageal fistula (TEF). After identification of the deep tracheal rings of the neck and chest, an endotracheal tube was placed through these rings allowing for ventilation at the level of the carina. Throughout the procedure, ventilation of the patient remained difficult with associated bradycardia and hypoxia necessitating 45 total minutes of CPR. Discussion between the Cardiothoracic Surgery service and Pediatric Surgery service determined that the patient would not qualify for extracorporeal membrane oxygenation (ECMO) and given the family’s goals of care, the decision was made to secure the airway without further intervention or management of the tracheoesophageal fistula. During the procedure, a chest X-ray (Figure 1) and head ultrasound were obtained. The chest X-ray was taken at the beginning of the procedure and the endotracheal tube is in place, presumably in the esophagus. The Head Ultrasound demonstrated a low anterior cerebral artery (ACA) resistive index, concerning for ischemia, and mildly echogenic periventricular white matter, concerning for white matter injury. Due to the laryngeal atresia, TEF, prematurity, cardiac defect, and critical decompensation, the patient’s poor prognosis was conveyed to the parents by the joint treatment team. Parents elected to proceed with palliative care to spend time with their child. An autopsy was performed at the request of the parents which revealed microscopic TEF. Given the atretic airway above the tracheostomy site and the minimal TEF, the method of ventilation following confirmed esophageal intubation remains largely unknown.

## Discussion

Laryngeal atresia (LA) is a rare, frequently fatal form of CHAOS wherein recanalization fails to occur during the development of the larynx in the tenth week of gestation. In the landmark paper describing CHAOS, four fetuses were evaluated, three of which were found to have coexistent fetal anomalies, none of which survived [1]. While this description of CHAOS has aided in prenatal diagnosis and early intervention in some cases, not all cases of CHAOS will result in these findings. CHAOS is frequently comorbid with other congenital anomalies which can obscure typically described ultrasound findings [4]. In particular, the presence of tracheoesophageal fistula (TEF), as in this case, can allow for the exit of pulmonary secretions thereby permitting normal lung development and resulting in the absence of traditional CHAOS ultrasound findings [4]. As a result, attempts have been made to adjust definitions of CHAOS to be more inclusive of such presentations. In 2002, Hartnick et al. described three distinct categories of CHAOS (Table 1), only one of which would present with classic prenatal ultrasound findings [5].

**Table 1:** CHAOS can be subcategorized depending on the degree of obstruction and the presence or absence of TEF CHAOS: Congenital high airway obstruction syndrome; TEF: Tracheoesophageal fistula; LA: Laryngeal atresia

Type	Description
Type 1	Complete LA without an esophageal fistula
Type 2	Complete LA with TEF
Type 3	Near-complete high upper airway obstruction

Without treatment, LA is universally fatal and as such there are distinct advantages to prenatal diagnosis including time to carefully plan and prepare for a safe delivery. In children diagnosed with CHAOS in utero, one option for treatment is the ex utero intrapartum treatment (EXIT) [6]. This procedure involves partial delivery of the fetus with maintenance of the uteroplacental circulation during fetal airway securement through intubation or surgery. While EXIT is an excellent option for some fetuses diagnosed with CHAOS in utero, limitations do exist. Notably, increases in lung distention, eversion of diaphragms, and worsening ascites throughout development as a direct result of airway obstruction can result in spontaneous abortion before the fetus reaches a gestational age compatible with life [7]. In such cases, fetoscopic balloon dilation and cricotracheal resection have been successfully performed to allow for EXIT and tracheostomy at an appropriate gestational age. While such advances in technology are incredibly helpful for those receiving adequate prenatal care who exhibit classic CHAOS findings, many cases of LA are discovered at delivery and therefore require a high index of clinical suspicion. Typical findings in infants born with untreated LA include an absence of audible cry despite crying effort, hypoxia despite adequate respiratory effort, and failure to intubate [8]. In such cases airway management becomes emergent, frequently requiring immediate surgical airway management with tracheostomy when intubation cannot be performed. Such a decision must be made quickly due to increasing hypoxemia and known association between multiple failed intubation attempts and adverse outcomes [9].

While tracheoesophageal fistula is the most common comorbid congenital anomaly in patients with LA, it is far from the only anomaly. In fact, LA is comorbid with numerous other congenital anomalies. In one study of 21 patients, 19 of these (90%) had additional associated malformations [10]. While a multitude of malformations were described in addition to TEF (the most common anomaly comorbid with LA), additional comorbid findings included imperforate anus, genital malformations, vesical anomalies, bilateral renal agenesis/dysplasia, horseshoe/dysplastic kidneys, lung segmentation anomalies, facial dysmorphia, and a variety of cardiac anomalies. While no specific genetic causes have been linked to laryngeal atresia, it has been shown to occur in conjunction with some genetic conditions including 22q11 deletion syndrome [11]. More research is needed to determine if any specific genetic mutations cause laryngeal atresia. Given how often laryngeal atresia presents with associated congenital anomalies, their presence should raise the index of suspicion for laryngeal atresia in difficult to ventilate/oxygenate newborns.

## Conclusions

While laryngeal atresia is a rare condition, missed diagnosis and failure to initiate prompt treatment is universally fatal. Due to advances in maternal-fetal medicine, prenatal diagnosis has allowed for survival in some patients with LA. However, these interventions rely on traditional prenatal ultrasound findings of CHAOS which are found only in a subset of patients. As a result, many patients with LA experience unexpected hypoxia and difficulty with intubation at birth creating an emergent airway management challenge with a high degree of morbidity and mortality. This case seeks to highlight typical findings in patients with LA and to reiterate the strong association between LA and additional congenital anomalies which should raise the index of suspicion and aid in rapid, appropriate airway management. More research is needed to determine appropriate methods of prenatal diagnosis in cases where classic CHAOS findings are absent in order to improve outcomes in these patients.
